# Associations of individual and joint expressions of ERCC6 and ERCC8 with clinicopathological parameters and prognosis of gastric cancer

**DOI:** 10.7717/peerj.11791

**Published:** 2021-07-15

**Authors:** Jing Chen, Liang Li, Liping Sun, Yuan Yuan, Jingjing Jing

**Affiliations:** 1 Tumor Etiology and Screening Department of Cancer Institute and General Surgery, the First Hospital of China Medical University, Shenyang, Liaoning, China; 2 Key Laboratory of Cancer Etiology and Prevention in Liaoning Education Department, the First Hospital of China Medical University, Shenyang, Liaoning, China; 3 Key Laboratory of GI Cancer Etiology and Prevention in Liaoning Province, the First Hospital of China Medical University, Shenyang, Liaoning, China

**Keywords:** Gastric cancer, Prognosis, Clinicopathological parameters, ERCC6, ERCC8, Expression

## Abstract

**Background:**

Excision repair cross-complementing group 6 and 8 (ERCC6 and ERCC8) have been implicated in ailments such as genetic diseases and cancers. However, the relationship between individual and joint expressions of ERCC6/ERCC8 and clinicopathological parameters as well as prognosis of gastric cancer (GC) still remains unclear.

**Methods:**

In this study, protein expressions of ERCC6, ERCC8 and ERCC6-ERCC8 were detected by immunohistochemistry (IHC) in 109 paired GC and para-cancerous normal tissue samples. The mRNA expression was detected in 36 pairs of tissue samples. IHC results and RNA-seq data extracted from The Cancer Genome Atlas (TCGA) were used to explore the clinical value of ERCC6 and ERCC8 expression in GC. We further conducted protein-protein interaction analysis, Gene Ontology, Kyoto Encyclopedia of Genes and Genomes, gene set enrichment analysis, and gene-gene interaction analysis to explore the function and regulation networks of ERCC6 and ERCC8 in GC.

**Results:**

Individual and joint ERCC6/ERCC8 expression were significantly higher in adjacent normal mucosa compared with GC tissues. ERCC6 mRNA expression showed no difference in GC and paired tissues, while ERCC8 mRNA was significantly decreased in GC tissues. Protein expression of ERCC6, ERCC8, double negative ERCC6-ERCC8 and double positive ERCC6-ERCC8 and overexpressed ERCC6 mRNA were related to better clinicopathologic parameters, while overexpressed ERCC8 mRNA suggested worse parameters. Univariate survival analysis indicated that the OS was longer when ERCC6 protein expression and ERCC8 mRNA expression increased, and double negative ERCC6-ERCC8 expression was associated with a short OS. Bioinformatics analyses showed ERCC6 and ERCC8 were associated with nucleotide excision repair (NER) pathway, and six and ten gene sets were figured out to be related with ERCC6 and ERCC8, respectively. KEGG pathway showed that ERCC6/ERCC8 related gene sets were mainly involved in the regulation of PI3K/AKT/mTOR pathway. Direct physical interactions were found between ERCC6 and ERCC8.

**Conclusions:**

Individual and joint expressions of ERCC6/ERCC8 were associated with clinical features of GC. Protein expression of ERCC6, ERCC6-ERCC8, and mRNA expression of ERCC8 were related to prognosis of GC. ERCC6 and ERCC8 primarily function in the NER pathway, and may regulate GC progression through the regulation of PI3K/AKT/mTOR pathway.

## Introduction

The human genome is under a condition where DNA impairment and correction are in a dynamic equilibrium. A variety of DNA repair mechanisms have been found to help maintain the integrity of genes damaged by endogenous and exogenous variables (Lindahl & Wood, 1999). Nucleotide excision repair (NER) can repair a wide range of DNA lesions, including oxidatively damaged DNA bases, bulky adducts and UV-induced cyclobutane pyrimidine dimers (Marteijn et al., 2014; Cleaver, Lam & Revet, 2009). The repair pathway cannot be activated when DNA repair gene expression is deficient, which can lead to a decrease of DNA repair capacity and an increase of cancer susceptibility (O’Driscoll, 2012).

Excision repair cross-complementing group 6 (ERCC6/CSB) and excision repair cross-complementing group 8 (ERCC8/CSA) are core members vital for NER pathway (Hanawalt & Spivak, 2008; Enoiu, Jiricny & Schärer, 2012; Iyama et al., 2015; Vermeulen & Fousteri, 2013). Emerging evidence indicated that ERCC6 and ERCC8 functioned in the same biological pathways and sub-pathways (Boetefuer, Lake & Fan, 2018; Bradsher et al., 2002). Considering their important function in the NER pathway, many scholars conducted experiments to explore the roles of ERCC6 and ERCC8 in disease onset and progression. Results of these experiments showed that the expression of ERCC6 and ERCC8 had clinical significance in ailments such as genetic diseases and cancers. Cheng et al. (2000) and Cheng et al. (2002) observed that patients with lung cancer and head and neck cancer were more likely to have decreased ERCC6 mRNA expression in comparison with controls. In contrast, overexpressed ERCC6 mRNA was detected in colorectal cancer compared with matched normal tissues (Yu et al., 2006). Caputo et al. (2013) also reported higher ERCC6 mRNA expression in renal cell carcinoma and lung cancer samples. Their western blot results revealed increased ERCC6 protein levels in bladder, cervix, prostate and breast cancer cells compared with normal cells. Previous researches have also revealed that ERCC6 and ERCC8 played an important role in the occurrence and progression of a hereditary disease named Cockayne syndrome (Friedberg, 2001). One study performed by Moslehi had implicated the potential interaction of ERCC6 with ERCC8 in breast cancer susceptibility (Moslehi et al., 2020). Another research suggested that cells with mutations in ERCC6 and ERCC8 were sensitive to reactive oxygen species, which was one of the critical relevant mechanisms of cancers (Carbone et al., 2020). It has also been observed in our previous study that ERCC6, ERCC8, and ERCC6-ERCC8 joint expression is related to the risk of gastric cancer (GC) (Jing et al., 2017).

All current data indicate that there exists heterogeneous expression of ERCC6 and ERCC8 in disease, thus influencing the development and progression of diseases. However, to date no prior report has reported the effect of ERCC6, ERCC8 and ERCC6-ERCC8 expression on the prognosis of GC. For the first time, our study conducted a comprehensive analysis using immunohistochemistry (IHC) and RNA-seq data to explore the associations between ERCC6 and ERCC8 expression and clinicopathological parameters and prognosis of GC.

## Materials & Methods

### Human tissue specimens

Paired gastric cancer and para-cancerous normal tissue samples were collected from 145 individuals with gastric cancer, who were diagnosed at the anorectal department of the First Hospital of China Medical University. Each selected patient had not received neoadjuvant chemoradiotherapy or any other treatment from 2012 to 2015. Histological diagnoses were completed following the updated Sydney System Classification (Stolte & Meining, 2001) and the World Health Organization criteria (Hamilton & Aaltonen, 2000) for gastritis and GC, respectively. The 2010 7th edition of the TNM staging system of the International Union Against Cancer/American Joint Committee on Cancer was selected to stage the tumor (Santiago, Sasako & Osorio, 2009), based on postoperative pathologic examination. This study was approved by the ethics committee of the First Hospital of China Medical University. Written informed consent was obtained from all participants.

### Immunohistochemistry staining and evaluation

Every single paraffin-embedded sample was sectioned into a 4-µm-thick slide. All the deparaffinized and rehydrated slides were boiled at 95 °C for 30 min in citrate antigen retrieval solution (pH 6.0). Immunohistochemistry (IHC) staining was performed with the avidin-biotin complex method as our previous study described (Jing et al., 2017). CSB (ERCC6) rabbit polyclonal antibody (1:300 dilution, Origene, TA313375, USA) and AB1(anti-ERCC8) antibody produced in rabbit (1:500 dilution, Sigma, AV31542, USA) were used.

Two experienced pathologists who were blinded to patient-related information evaluated and scored the staining results independently. A semi-quantitative scoring criterion was applied to analyze the staining extent of each slide (Detre, Jotti & Dowsett, 1995). The staining score was categorized on the basis of intensity: 0 - no staining, 1 - light staining, 2 - moderate staining and 3 - strong staining and coloring ratio: 0 (≤5%), 1 (5%–25%), 2 (25%–50%), 3 (50%–75%), 4 (≥75%) of the IHC results. An immunoreactivity score (IS) was generated by multiplying the two scores for each sample. All the scores were applied to indicate certain extent of positive staining except the score of 0, which suggested a negative protein expressed level.

### RNA isolation and qRT-PCR

Total RNA were extracted from 36 pairs of tissue specimens with RNAiso Plus reagent based on the experimental guidelines (TaKaRa, Kusatsu, Japan). The cDNA was synthesized by PrimeScript RT Master Mix following the protocol (Perfect Real Time; TaKaRa, Kusatsu, Japan). Relative expressions of ERCC6 and ERCC8 were detected with TB Green Premic EX Taq II (TliRNaseH Plu; TaKaRa, Kusatsu, Japan). β-actin was selected as the endogenous reference control. Each melting curve was with single peak in the study. A 2−ΔCt method was applied to assess the relative expression levels of ERCC6 and ERCC8. Primers for ERCC6, ERCC8 and β-actin were tabulated in Table S1.

### Clinical information and RNA-seq data collection

Clinical information of 109 IHC cases concerning age, gender, smoking, family history and alcohol consumption were obtained via questionnaire. Clinical characteristics were collected from medical records, including TNM stage, Lauren’s classification, Borrmann classification, tumor size, phase of progression, lymph node metastasis, perineural invasion, and vascular invasion. The final follow-up was completed on July 2016. Full data concerning prognosis were obtained from 97 participants.

Data of 415 stomach cancer patients, which included RNA-seq data and survival information, were downloaded from TCGA (https://cancergenome.nih.gov/) (Weinstein et al., 2013; Akbani et al., 2014). Detailed information of clinicopathological parameters including age, gender, grade, TNM stage and histological type were also downloaded for further analyzing. GEPIA (Gene Expression Profiling Interactive Analysis) (http://gepia.cancer-pku.cn/) was applied to obtain the mRNA expression levels of ERCC6 and ERCC8 and the data of disease-free survival (DFS) (Tang et al., 2017).

### Interaction and functional analysis of ERCC6 and ERCC8

Given the clinical significance of ERCC6/ERCC8 in GC, we further investigated their biological functions. First we used ERCC6 and ERCC8 as core genes to construct protein-protein interaction (PPI) networks by Search Tool for the Retrieval of Interacting Genes (STRING v.11.0; https://string-db.org/; accessed on August 27, 2020), to mine proteins that have functional interactions with ERCC6/ERCC8 (Szklarczyk et al., 2019). Analytic information including nodes degrees and biological networks was visualized with Cytoscape platform (v.3.7.2) (Su et al., 2014). And ten most associated proteins were showed in the diagrams.

Then we conducted enrichment analyses of Gene ontology (GO) and Kyoto Encyclopedia of Genes and Genomes (KEGG) to explore the biological functions of ERCC6 and ERCC8 with the Database for Annotation, Visualization and Integrated Discovery (DAVID; v.6.8; https://david.ncifcrf.gov/home.jsp; accessed on August 31, 2020), a user friendly database providing comprehensive analysis of gene annotation (Huang da, Sherman & Lempicki, 2009). R language (Version 3.6.3) and the ggplot2 package were applied to visualize the analytic results. Terms with a *P* < 0.05 were deemed significant and for GO, only top ten terms of each group were selected to be visualized.

### Identification of regulation networks of ERCC6 and ERCC8 by GSEA /KEGG

Gene set enrichment analysis was conducted on the GSEA platform (version 4.1.0; https://www.broadinstitute.org/gsea/) coupled with MSigDB database (Subramanian et al., 2005a; Subramanian et al., 2005b). Oncogenic signature gene sets (c6.all.v7.1.symbols.gmt) and TCGA expression data were included in this analysis. Through C6 Oncogenic Signatures of GSEA, we could clarify the gene sets associated regulation networks that were involved in ERCC6 and ERCC8. First TCGA expression data was grouped into ERCC6/ERCC8-high and ERCC6/ERCC8-low groups according to their expression levels. And then differentially expressed genes of the two groups were figured out. Finally significantly changed oncogenic regulation networks of these genes were identified through a thousand times of phenotype permutation test and the metric for the analysis was set as pearson. A normalized *p* value <0.01 and a false discovery rate (FDR) less than 0.25 were selected as criteria for significant enrichment results. Normalized enriched score (NES) was applied to rank the obtained results. KEGG pathway analysis was further conducted to identify pathways these gene sets mainly involved in.

### Identifying gene-gene interactions between ERCC6 and ERCC8

To identify interactions between ERCC6 and ERCC8, we performed gene-gene interaction analysis with the Gene Multiple Association Network Integration Algorithm (GeneMANIA; https://www.genemania.org/; accessed on November 13, 2020), which is a user-friendly interface providing analysis with available genomics and proteomics data (Warde-Farley et al., 2010). To display interactions, nodes and links represented genes and networks respectively in the visualized results.

### Validation of protein expression of ERCC6/ERCC8 related genes

CCLE (The Broad Institute Cancer Cell Line Encyclopedia; http://www.broadinstitute.org/ccle) is an online database which provides genomic data and protein expression of more than 900 cancer cell lines (Barretina et al., 2012). To validate the protein expression of the genes obtained from GSEA, we extracted mRNA expression data of ERCC6, ERCC8 and protein expression data of EIF4E, ERBB2, JAK2, Src, and Cyclin_D1, which were mainly involved in PI3K/AKT/mTOR pathway, from 37 gastric cancer cell lines. Pearson’s correlation was further calculated to evaluate the correlation between their expression levels. A *P* < 0.05 was deemed significant.

### Statistical analysis

IBM SPSS Statistics for Windows, version 23.0 (IBM Corp., Armonk, N.Y., USA), GraphPad Prism V5.0 software (GraphPad software, USA) and R platform (Version 3.6.3) were used for statistical analyses. For IHC, Pearson *χ*^2^ test was used when analyzing the correlations between ERCC6 and ERCC8 expressed levels and clinicopathological parameters; univariate and multivariate Cox regression analyses have been selected for the determination of their impact on overall survival (OS), and variables including age, TNM stage, perineural invasion, vascular invasion and lymph node metastasis were further adjusted in the multivariate model to evaluate the independent prognostic value. For qRT-PCR data, Wilcoxon matched-pairs signed rank test was used to assess the differential expression of ERCC6 and ERCC8 among different groups. For RNA-seq data, Wilcoxon test and Kruskal-Wallis H test have been employed when calculating the interrelationships between ERCC6/8 expressions and clinical characteristics; two-sided Log-rank test and multivariate Cox proportional-hazards model adjusted by gender, age, grade, stage, T, N, and M were selected to clarify the prognostic value of ERCC6 and ERCC8. A *P* < 0.05 suggested statistical difference.

## Results

### Expression of ERCC6 and ERCC8 in GC and adjacent normal mucosa

**Figure 1:**
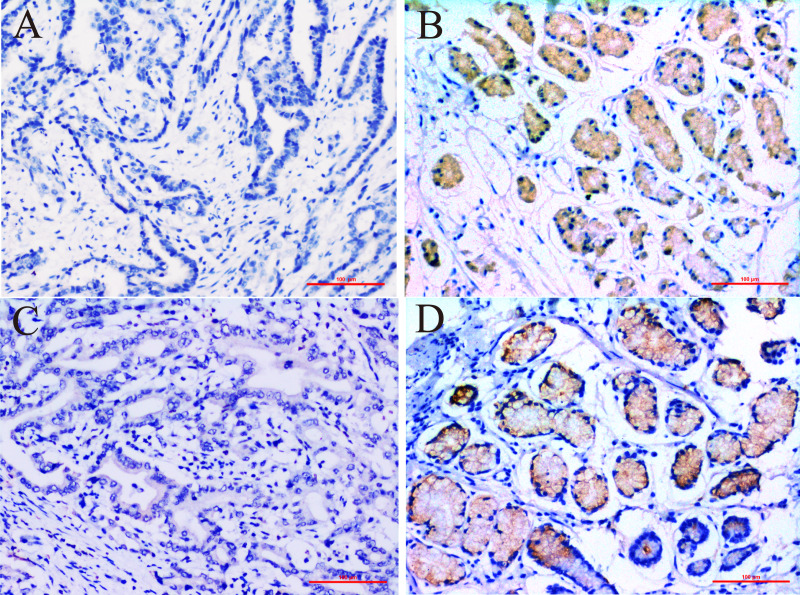
Immunohistochemical staining for ERCC6 and ERCC8 expression in GC and its paired pan-cancerous normal tissues. (A) GC with negative ERCC6 expression; (B) paired pan-cancerous normal tissues with positive ERCC6 expression; (C) GC with negative ERCC8 expression; (D) paired pan-cancerous normal tissues with positive ERCC8 expression. (Magnification ×200; bar = 100 µm).

**Figure 2:**
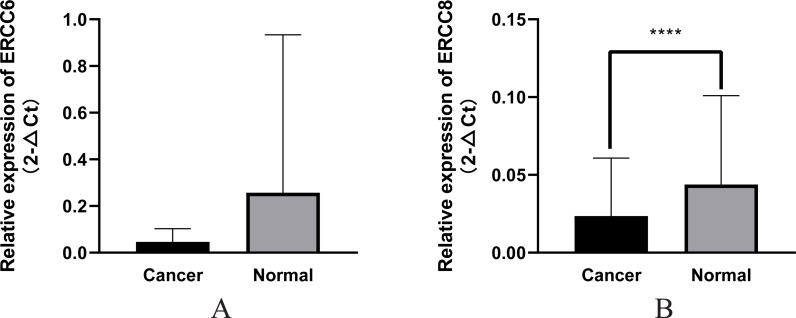
Relative mRNA expression of ERCC6/ERCC8 in GC and paired normal tissues. (A) ERCC6; (B) ERCC8. Significant results * *P* < 0.0001).

In this study we compared the expressed levels of ERCC6 and ERCC8 between GC and adjacent normal mucosa. Representative ERCC6 and ERCC8 staining were present in Fig. 1. Our results suggested that individual and joint expressions of ERCC6 and ERCC8 were obviously higher in adjacent normal mucosa than in GC tissues (all *P* < 0.001) (Table 1). Specifically, the ERCC6-ERCC8 double positive rate dropped to 16.5% in GC and the double negative rate was only 1.8% in adjacent normal mucosa. As shown in Fig. 2, no significant difference was observed with ERCC6 mRNA expression in GC (*P* = 0.300) and ERCC8 mRNA expression was lower in GC tissues when compared with normal tissues (*P* < 0.0001).

**Table 1:** Protein expression of ERCC6/ERCC8 in different gastric tissues.

	**Adjacent SG vs GC**							
	**ERCC6**		**ERCC8**		ERCC6-ERCC8^*^		ERCC6-ERCC8^**^	
**Positive (%)**	105(96.3)	44(40.4)	104(95.4)	42(38.5)	102(93.6)	18(16.5)	107(98.2)	60(55.0)
**Negative (%)**	4(3.7)	65(59.6)	5(4.6)	67(61.5)	7(6.4)	91(83.5)	2(1.8)	49(45.0)
**P**	**<0.001**		**<0.001**		**<0.001**		**<0.001**	

**Notes.**

GC gastric cancer SG superficial gastritis

* Double positive for ERCC6 and ERCC8 expression.

** Double negative for ERCC6 and ERCC8 expression.

### Correlations of ERCC6 and ERCC8 expression with clinicopathological characteristics in GC

We explored the associations of ERCC6/ERCC8 expressed levels with clinicopathological parameters in GC patients and the results were summarized in Table S2. ERCC6 expression was significantly related to Borrmman classification (*P* = 0.017), Lauren’s classification (*P* = 0.004) , TNM stage (*P* = 0.005) (*P* = 0.012 for T stage) and perineural invasion (*P* = 0.001). High ERCC6 expression was observed in gastric cancers of Borrmman class I–II, TNM stage I–II, intestinal-type and without perineural invasion. Expression of ERCC8 was statistically higher in patients with TNM stage I-II in comparison to stage III-IV (*P* < 0.001) (*P* < 0.001 for T stage; *P* = 0.002 for lymph node metastasis), and was higher in those with early-stage and small size (*P* = 0.031 and 0.007, respectively). Higher expression of ERCC8 was also observed in intestinal type GC (*P* = 0.008). As for the joint expression of ERCC6/ ERCC8, double positivity was related to small tumor size (*P* = 0.005), Borrmman I-II stage (*P* < 0.001), TNM I-II stage (*P* = 0.001) (*P* < 0.001 for T stage), Lauren intestinal type (*P* = 0.014) of GC and negative perineural invasion (*P* = 0.015). Double negativity was associated with TNM III–IV stage (*P* < 0.001) (*P* = 0.002 for T stage), positive perineural invasion (*P* = 0.002), advanced stage (*P* = 0.034) and diffuse type (*P* < 0.001) of GC.

**Figure 3:**
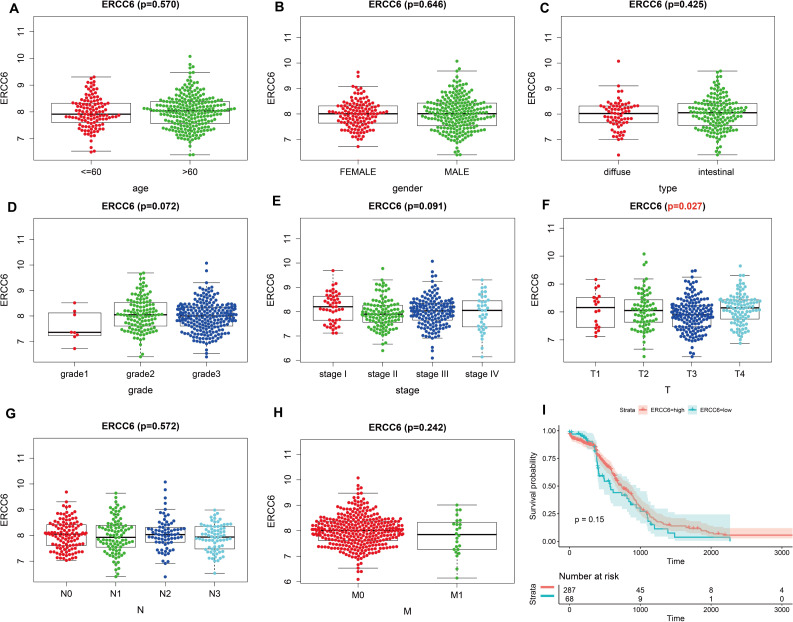
Correlation analysis of ERCC6 expression with clinicopathological parameters and survival of GC using TCGA data. (A) Age, (B) gender, (C) type, (D) grade, (E) stage, (F) T stage, (G) N stage, (H) M stage, (I) univariate survival analysis. A *P* < 0.05 was deemed statistically significant.

**Figure 4:**
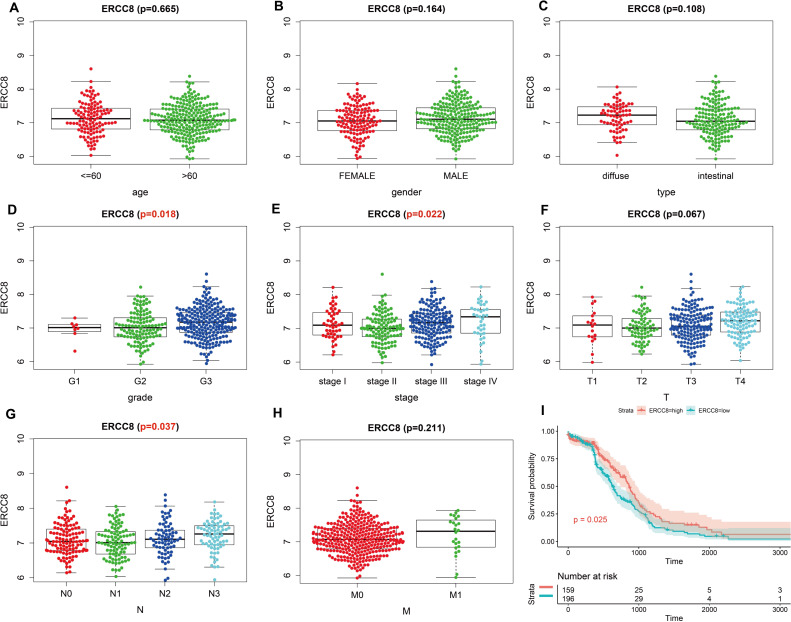
Correlation analysis of ERCC8 expression with clinicopathological parameters and survival of GC using TCGA data. (A) Age, (B) gender, (C) type, (D) grade, (E) stage, (F) T stage, (G) N stage, (H) M stage, (I) univariate survival analysis. A *P* < 0.05 was deemed statistically significant.

Analysis results of RNA-seq data obtained from TCGA was shown in Fig. 3, higher ERCC6 expression was related with better T stage (*P* = 0.027), which is consistent with the IHC analysis results, while no statistical results were found between ERCC6 expression and age (*P* = 0.570), gender (*P* = 0.646), histological type (*P* = 0.425), grade (*P* = 0.072), stage (*P* = 0.091), N stage (*P* = 0.572) and M stage (*P* = 0.242). As for ERCC8, Fig. 4 revealed that overexpressed ERCC8 was closely related to worse grade (*P* = 0.018), advanced stage (*P* = 0.022), and worse N stage (*P* = 0.037); the associations between ERCC8 expression and age, gender, histological type, T stage and M stage were not significant (all *P* > 0.05).

### The relationship between ERCC6 and ERCC8 expression and prognosis of GC

As shown in Table 2, univariate survival analysis showed no significant correlation between protein expressed levels of ERCC8 and GC prognosis (*P* = 0.211), while a significant correlation was observed between ERCC6 protein expressed levels and GC prognosis (*P* = 0.047, HR =3.416, 95% CI [1.017–11.475]). In addition, double negative expression of ERCC6 and ERCC8 was significantly associated with poorer prognosis (*P* = 0.022, HR = 2.603, 95% CI [1.148–5.905]). However, no statistical association was found between the expression levels of ERCC8/ ERCC6-ERCC8 and GC prognosis (both *P* > 0.05). Because TNM stage (*P* < 0.001), age (*P* = 0.039), perineural invasion (*P* = 0.043), vascular invasion (*P* = 0.007), and lymph node metastasis (*P* < 0.001) are all statistically associated with gastric cancer prognosis (Table S3), Cox’s proportional hazards model adjusted by perineural invasion, TNM stage, age and vascular invasion was further applied to evaluate the prognostic value. However, the multivariate analysis suggested that ERCC6 or ERCC8 or ERCC6-ERCC8 expressed level was not an independent factor for GC prognosis (all *P* > 0.05) (Table 2).

**Table 2:** Correlation between ERCC6/ERCC8 expression and survival in gastric cancer.

	**Case**	**Cases of events**	**MST**	**Univariate**			**Multivariate**		
				**P**	**HR**	**95% CI**	**P**	**HR**	**95% CI**
**ERCC6 expression**									
Positive	31	3	37.4		1(ref)			1(ref)	
Negative	66	22	31.1	**0.047**	**3.416**	**1.017–11.475**	0.284	2.034	0.556–7.444
**ERCC8 expression**									
Positive	40	9	35.9		1(ref)			1(ref)	
Negative	57	16	31.2	0.211	1.690	0.742–3.848	0.958	1.023	0.429–2.442
**ERCC6-8 expression**									
DP	16	3	36.2		1(ref)			1(ref)	
DN and SN	81	22	32.5	0.286	1.942	0.573-6.579	0.759	1.221	0.340–4.394
**ERCC6-8 expression**									
DP and SP	55	9	37.1		1(ref)			1(ref)	
DN	42	16	28.8	**0.022**	**2.603**	**1.148–5.905**	0.478	1.380	0.567–3.364

**Notes.**

GC gastric cancer MST median survival time HR hazard radio CI confidence interval ref. reference

Survival analysis with RNA-seq data suggested that higher ERCC8 mRNA expression was related to better OS (*P* = 0.025; Fig. 4I) while ERCC6 expression made no sense (*P* = 0.15; Fig. 3I). And multivariate Cox proportional hazard models showed no significant results with ERCC6 (*P* = 0.969; HR = 0.995; 95% CI [0.761 –1.300]) and ERCC8 (*P* = 0.078; HR = 1.393; 95% CI [0.964–2.014]). And the results suggested that double lower ERCC6 and ERCC8 mRNA expression was not significantly associated with GC prognosis neither in univariate model (*P* = 0.242; HR = 1.364; 95% CI [0.810–2.297]) nor in multivariate model (*P* = 0.197; HR = 1.426; 95% CI [0.832 –2.446]). There existed no significant correlation between ERCC6 mRNA expression (*P* = 0.600; File S4A), ERCC8 mRNA expression (*P* = 0.780; Supplementary file 4B) and DFS, respectively.

### Function analysis for ERCC6 and ERCC8

**Figure 5:**
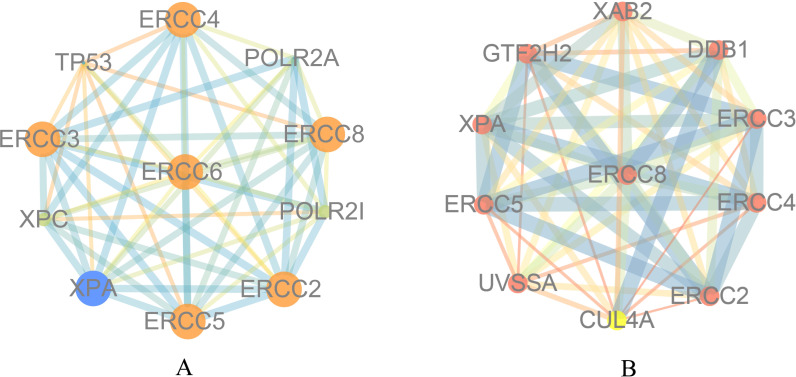
Protein interaction networks of 10 associated partners with a confidence score > 0.4 obtained from the String database. (A) ERCC6 is defined as the core gene; (B) ERCC8 is defined as the core gene.

To explore the function of ERCC6 and ERCC8, we first constructed PPI networks using ERCC6 and ERCC8 as core genes. With a confidence score of more than 0.4, Fig. 5A showed that ten most associated proteins of ERCC6 were ERCC2, ERCC3, ERCC4, ERCC5, ERCC8, TP53, XPC, XPA, POLR2A and POLR2I; and for ERCC8, the related proteins were XAB2, DDB1, ERCC3, ERCC4, ERCC2, CUL4A, UVSSA, ERCC5, XPA and GTF2H2 (Fig. 5B).

**Figure 6:**
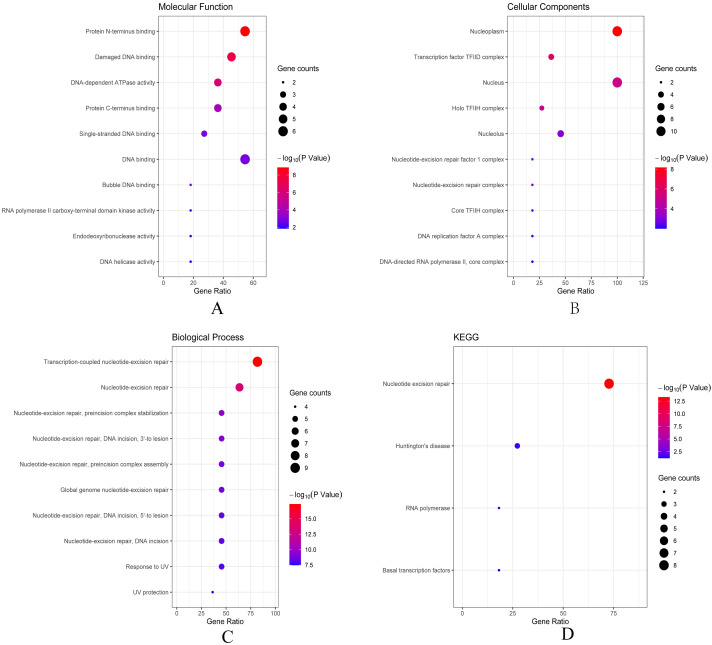
The bubble diagram of enrichment and pathway analysis of ERCC6 network genes. (A) Top ten categories for molecular function of GO analysis; (B) top ten categories cellular component of GO analysis; (C) top ten categories for biological process of GO analysis; (D) KEGG pathway analysis results.

**Figure 7:**
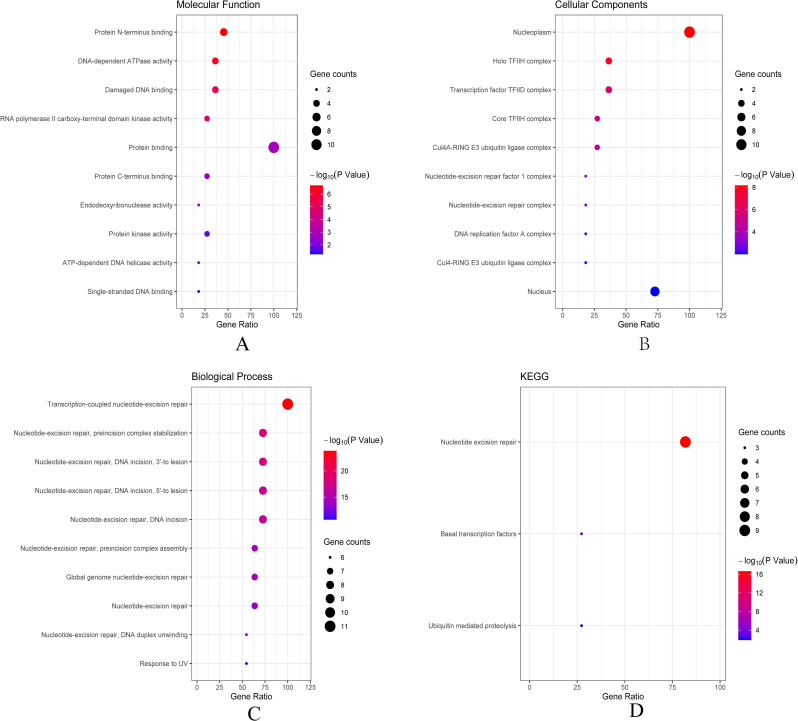
The bubble diagram of enrichment and pathway analysis of ERCC8 network genes. (A) Top ten categories for molecular function of GO analysis; (B) top ten categories cellular components of GO analysis; (C) top ten categories for biological process of GO analysis; (D) KEGG pathway analysis results.

Then we conducted analyses of GO and KEGG according to the network results we obtained from String. As revealed in Fig. 6, ERCC6 network genes showed enrichment in molecular functions of protein N-terminus binding, damaged DNA binding and DNA-dependent ATPase activity (Fig. 6A). They were mainly involved in nucleoplasm, transcription factor TFIID complex, and holo TFIIH complex according to cellular components analysis result (Fig. 6B). Figure 6C showed that ERCC6-interactive genes were significantly enriched in biological processes of nucleotide-excision repair and UV protection. Analysis results of KEGG suggested that these genes were closely related to nucleotide excision repair, Huntington’s disease, RNA polymerase and basal transcription factors (Fig. 6D). As for ERCC8 network genes, results of GO enrichment analysis showed that these genes were related to the composition of transcriptional initiation complexes and ubiquitin ligase complexes, and not surprisingly, their main molecular functions and participated biological processes bore a remarkable resemblance to ERCC6 network genes (Figs. 7A–7C). Similar KEGG results, nucleotide excision repair and basal transcription factors were also observed in ERCC8 network genes. And a special part of this analysis results was ubiquitin mediated proteolysis (Fig. 7D).

### Identification of gene sets associated regulatory networks of ERCC6 and ERCC8

**Figure 8:**
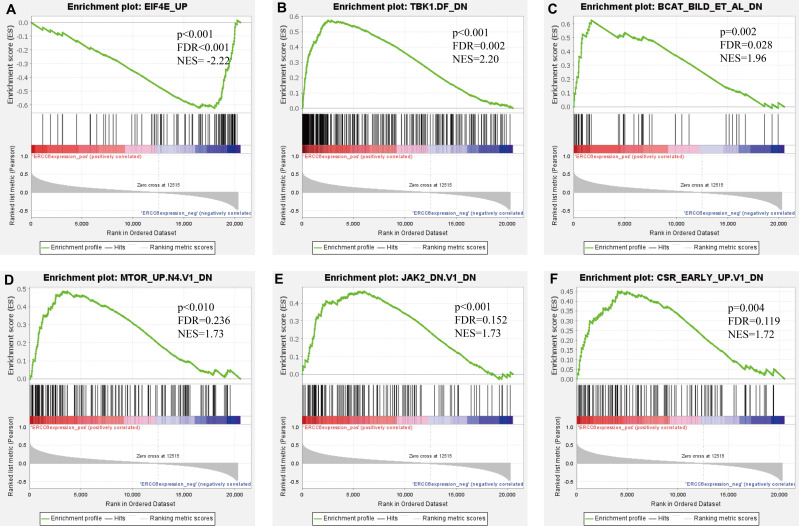
GSEA analyses results for ERCC6 in GC patients. GSEA results showing nine high-scoring gene sets including (A) EIF4E UP, (B) TBK1. DF DN, (C) BCAT BILD ET AL DN, (D) MTOR UP.N4.V1 DN, (E) JAK2 DN.V1 DN, (F) CSR EARLY UP.V1 DN are differentially enriched in ERCC6-related GC. NES, normalized enrichment score; FDR, false discovery rate.

**Figure 9:**
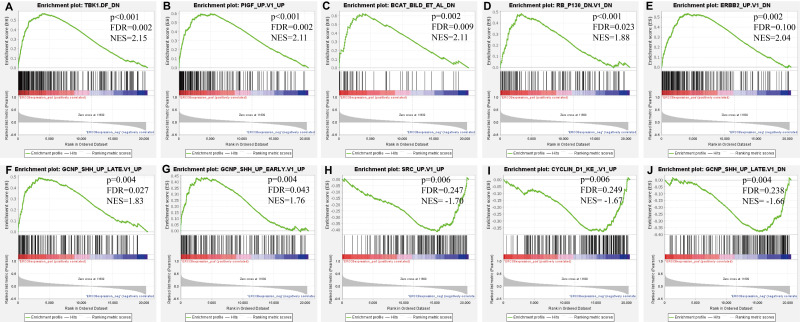
GSEA analyses results for ERCC8 in GC patients. GSEA results showing ten high-scoring gene sets including (A) TBK1. DF DN, (B) PIGF UP.V1 UP, (C) BCAT BILD ET AL DN, (D) RB P130 DN.V1 DN, (E) ERBB2 UP.V1 DN, (F) GCNP SHH UP LATE.V1 UP, (G) GCNP SHH UP EARLY.V1 UP, (H) SRC UP.V1 UP, (I) CYCLIN D1 KE.V1 UP and (J) GCNP SHH UP LATE.V1 DN are differentially enriched in ERCC8-related GC. NES, normalized enrichment score; FDR, false discovery rate.

Further, we identified the most positive and negative related gene sets with ERCC6 and ERCC8, to figure out the cellular regulatory networks in GC that ERCC6 and ERCC8 were involved in. Oncogenic signatures analysis indicated that in GC there existed six and ten most significant gene sets for ERCC6 and ERCC8, respectively. Among the results, both ERCC6 and ERCC8 were associated with TBK1 and BCAT associated cellular regulatory networks; ERCC6 was also associated with EIF4E, MTOR, JAK2 and CSR related regulatory networks; ERCC8 was also associated with PIGF, RB, ERBB2, GCNP, SRC and CYCLIN D1 related regulatory networks. Detailed information were shown in Fig. 8 for ERCC6 and Fig. 9 for ERCC8. KEGG pathway further revealed that these gene sets were mainly involved in the PI3K/AKT/mTOR pathway (File S5).

### Gene-gene interaction network between ERCC6 and ERCC8

**Figure 10:**
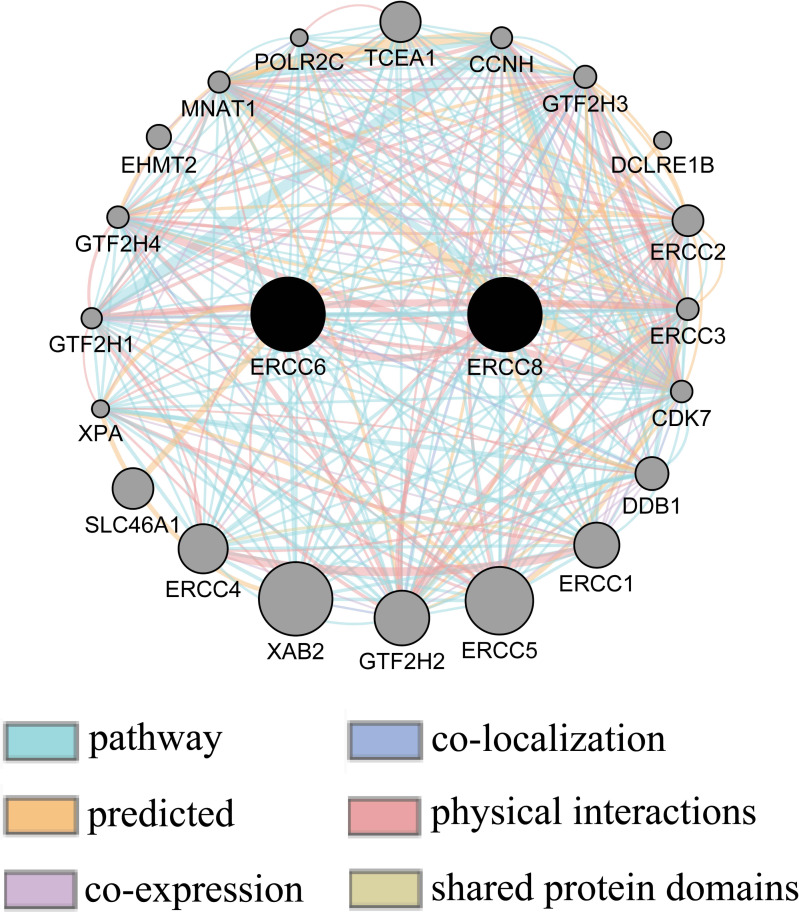
Gene-gene interaction network between ERCC6 and ERCC8. Nodes and links represent genes and networks, respectively.

Gene-gene interaction network accessed from GeneMANIA clarified the correlations of ERCC6 and ERCC8 among pathway, predicted, shared protein domains, physical interactions, co-localization, and co-expression. As shown in Fig. 10, there existed direct interactions including physical interactions and pathway and indirect interactions including prediction, co-expression, colocalization and shared protein domains between ERCC6 and ERCC8.

### Validation of protein expression of ERCC6/ERCC8 related genes in gastric cancer cell lines

Pearson’s correlation analysis using data from 37 gastric cancer cell lines showed that ERCC6 mRNA expression was significantly correlated with JAK2 protein expression (r = −0.345; *P* = 0.037), and ERCC8 mRNA expression was associated with Src protein expression (r = −0.417; *P* = 0.010). No significant relationship was found between ERCC6 mRNA expression and protein expression of EIF4E (r = −0.069; *P* = 0.686), ERBB2 (*r* = 0.295; *P* = 0.076), or between ERCC8 mRNA expression and Cyclin_D1 protein expression (*r* = 0.171; *P* = 0.311). Detailed information were shown in Table S4.

## Discussion

By analyzing IHC and TCGA data, our experiment elucidated that abnormally expressed ERCC6 and ERCC8 were associated with clinicopathological behaviors and survival of GC. Furthermore, by performing bioinformatics analysis of GO, KEGG, GSEA and gene-gene interaction analysis, our research extended the existing knowledge of ERCC6/ERCC8 in GC.

We initially detected protein and mRNA expressed levels of ERCC6/ERCC8 in GC and para-cancerous tissues. The results indicated that both individual and joint expression of these two genes were significantly decreased in GC in comparison to adjacent tissues. However, only ERCC8 mRNA expression in GC was significant different when compared with normal tissues. Then we investigated associations between protein expression of ERCC6 and ERCC8 and clinicopathological parameters, and we found that overexpressed ERCC6, ERCC8 and ERCC6-ERCC8 were significantly related to favorable clinicopathological features, which are key factors that have great impact on disease progression. RNA-seq data revealed that higher ERCC6 expression was associated with favorable T stage, while overexpressed ERCC8 was associated with unfavorable clinicopathological parameters. We suspected that the discrepancy may be due to some potential mechanisms that resulted in the instability of ERCC8 protein in GC progression. A recent research reported that ERCC6 deficiency could result in heterochromatin loss and exacerbates cellular aging (Lee et al., 2019). Defects in ERCC6 and ERCC8 will influence the coupling of transcription and repair to a certain extent, thus leading to declining DNA repair capacity (Venema et al., 1990). Physiologically, DNA repair capacity could be related to expression levels of proteins involved in DNA repair activities (Tuteja & Tuteja, 2001). Cancer cells lacking ERCC6 or ERCC8 protein, which are responsible for DNA repair, may exhibit a more malignant and poorly differentiated phenotype. Previous studies have also reported that a downregulation of DNA repair genes is related to late stage cancers and malignant transformation (Ganzinelli et al., 2011). Therefore, it is conceivable that the expression status of DNA repair genes could reflect the capacity of a cell to meet repair demands after being stimulated by a carcinogen. We suggested that ERCC6 and ERCC8 downregulation could induce persistent existence of unrepaired DNA lesions, decreased DNA repair capacity and increased cancer susceptibility, and eventually lead to cancer progression.

Further, to explore the prognostic value of ERCC6 and ERCC8, we investigated the correlation between ERCC6 and ERCC8 expression and survival in GC patients using IHC as well as RNA-seq data. According to univariate survival analysis based on IHC, higher ERCC6 protein expression was associated with better prognosis while double negative ERCC6 and ERCC8 expression indicated worse overall survival of GC patients. RNA-seq data also showed that overexpressed ERCC8 was related to a better OS of GC patients. When adjusting for certain parameters in the Cox multivariate analysis, analyses results of ERCC6 and ERCC8 expression with IHC and RNA-seq data no longer maintained independent predictive power, which may be due to the complexity of tumor progression. A previous lab study showed that knockdown of ERCC6 could sensitize HCT116 cells to 5-Fluorouracil in xenograft mouse models and colorectal cancer patients with high ERCC6 expression exhibited shorter overall survival (Zhao, Zhang & Li, 2017). As for other DNA repair family genes, high ERCC5 expression was shown to correlate with shorter survival time compared with low ERCC5 expression (Deng et al., 2014a), whereas decreased ERCC1 expression was reported to predict a favorable prognosis in GC (Deng et al., 2014b). Generally, expression of genes can be divided into transcription level and translation level, of which the products are mRNA and protein respectively. It is acknowledged that protein expression could be regulated by post-transcriptional process including ubiquitination, methylation, acetylation and phosphorylation, and thus resulting in differential expression status of mRNA and protein. And finally, differential expression status at two levels showed different connection with clinicopathological parameters/prognosis. On this basis, we believed that the differential correlation of ERCC6/ERCC8 mRNA expression and protein expression with clinicopathological parameters could be attribute to the unparallel expression status. We suspected that there exists some regulatory mechanisms in the process of ERCC6/8 mRNA to ERCC6/8 protein which made the evaluation of prognostic value based on the two levels of ERCC6 expression is inconsistent. Overall, our data suggested that protein expression levels of ERCC6, ERCC6-ERCC8, and ERCC8 mRNA expression, to some extent, may possess potential prognostic value in GC, and some certain factors should also be taken into account to estimate GC prognosis more comprehensively in the further analysis.

Next, bioinformatic analyses were conducted to better investigate biological functions and regulation networks of ERCC6 and ERCC8 in GC progression. First we queried the 10 most relevant genes of ERCC6 and ERCC8 through String and then performed GO and KEGG analyses with the obtained results. Enrichment analysis of ERCC6 and ERCC8 and their relevant genes showed similar results. Both the two genes were mainly involved in the composition of transcriptional initiation complexes and exerted influences on diverse nucleotide excision repair pathways. Similarly in other experiments, researchers have identified ERCC6 and ERCC8 as core NER genes (Boetefuer, Lake & Fan, 2018; Fousteri & Mullenders, 2008; Pines et al., 2018). KEGG pathway analysis results further revealed that ERCC6 also functioned in Huntington’s disease and ERCC8 showed significant impacts in ubiquitin mediated proteolysis. Consistent with our analyses, one study have reported that ERCC8 was involved in the formation of a complex which exhibits ubiquitin ligase activity (Saijo, 2013). Furthermore, we conducted GSEA analysis to identify ERCC6/ERCC8 associated regulation networks in GC. Here in our study, GSEA analysis suggested that ERCC6 was significantly associated with the oncogenic signatures of EIF4E, TBK1, BCAT, mTOR, JAK2 and CSR related regulation networks and ERCC8 was related to TBK1, PIGF, BCAT, RB, ERBB2, GCNP, SRC and CYCLIN_D1 associated oncogenic regulation networks. Through analyzing the expression data of 37 gastric cancer cell lines from CCLE, we figured out that ERCC6 mRNA expression was correlated with JAK2 protein expression, and that ERCC8 mRNA expression was related to Src protein expression. More importantly, KEGG analysis with these genes furtherly illustrated that these genes mainly functioned in the PI3K/AKT/mTOR pathway. These days emerging evidence has illustrated that PI3K/AKT/mTOR pathway deregulation plays an important part in GC progression (Al-Batran, Ducreux & Ohtsu, 2012). Currently, one study conducted by Riquelme mentioned that two mTOR pathway genes, EIF4E and mTOR, were overexpressed in GC cells (Riquelme et al., 2016). It has been found that ERBB2 could mediate the activation of PI3K (Fan & Weiss, 2010). Moreover, some studies have reported the environment-dependent inhibition or activation role of TBK1 in mTOR signaling (Bodur et al., 2018; Cooper et al., 2017; Hasan et al., 2017). Another investigation has proved the crosstalk between JAK2 and mTOR in the regulation of colorectal cancer (Zhang et al., 2020). Fiskus observed in their study that HEL/TGR cells with high levels of p-JAK2 seemed to be addicted to the pro-survival and pro-growth signaling through PI3K/AKT/mTOR (Fiskus et al., 2013). JAK2^V 617F^ was reported to activate PI3K/AKT/mTOR pathway through mimic growth factor signaling (Machado-Neto et al., 2020). One exploration illustrated that Src could mediate PI3K/Akt/mTOR pathway to regulate autophagy of osteosarcoma cells (Zhao et al., 2018). Therefore, given all the above results, we suspected that ERCC6 and ERCC8 could regulate GC progression through the regulation of PI3K/AKT/mTOR pathway. Because of the similar and identical functions and pathways found in our analysis, we then did gene-gene interaction analysis to figure out the potential associations between ERCC6 and ERCC8. The results demonstrated that there did exist direct physical interactions and pathways between ERCC6 and ERCC8, which was supported by one previous study (Wu, Feng & Stein, 2010). Indirect interactions including prediction, co-expression, colocalization and shared protein domains were also revealed. These results suggested the existence of alliance mechanisms between ERCC6 and ERCC8, which needs further in-depth study.

## Conclusions

In conclusion, individual and joint expressions of ERCC6 and ERCC8 were associated with clinical features of GC. Protein expressed levels of ERCC6, ERCC6-ERCC8, and ERCC8 mRNA expression were related to prognosis of GC patients. ERCC6 and ERCC8 primarily function in the NER pathway, and may regulate GC progression through the regulation of PI3K/AKT/mTOR pathway. Direct physical interactions existed between ERCC6 and ERCC8. However, a larger cohort is in need for the validation of these conclusions, and the mechanisms underlying these results warrant further experimental investigations.

## Supplemental Information

10.7717/peerj.11791/supp-1 Supplemental Information 1 The list of primers of ERCC6/ERCC8/*β*-actin

10.7717/peerj.11791/supp-2 Supplemental Information 2 Association of ERCC6/ERCC8 expression with clinicopathological features in gastric cancer

10.7717/peerj.11791/supp-3 Supplemental Information 3 Clinicopathological parameters and survival in GC GC, gastric cancer; MST, median survival time.

10.7717/peerj.11791/supp-4 Supplemental Information 4 Disease free survival analysis results of RNA-seq data of ERCC6/ERCC8 A.ERCC6; B.ERCC8

10.7717/peerj.11791/supp-5 Supplemental Information 5 KEGG pathway results of ERCC6/ERCC8 related gene sets Source credit: Kanehisa M, Sato Y, Kawashima M, Furumichi M, Tanabe M. KEGG as a reference resource for gene and protein annotation. Nucleic acids research. 2016;44(D1):D457-62.

10.7717/peerj.11791/supp-6 Supplemental Information 6 Expression correlation between ERCC6/8 and related genes Pearson product-moment correlation coefficient; *P* < 0.05 was deemed significant

10.7717/peerj.11791/supp-7 Supplemental Information 7 Raw data of participant baseline characters and measurements

## Additional Information and Declarations

### Data Availability

The following information was supplied regarding data availability: The raw data and measurements are available in the Supplemental File.
