# Supplementary figures and images for: Associations of individual and joint expressions of ERCC6 and ERCC8 with clinicopathological parameters and prognosis of gastric cancer
Source: PeerJ. 2021 Jul 15;9:e11791. doi: 10.7717/peerj.11791 (PMC8286707; doi:10.7717/peerj.11791)

# Disease Free Survival

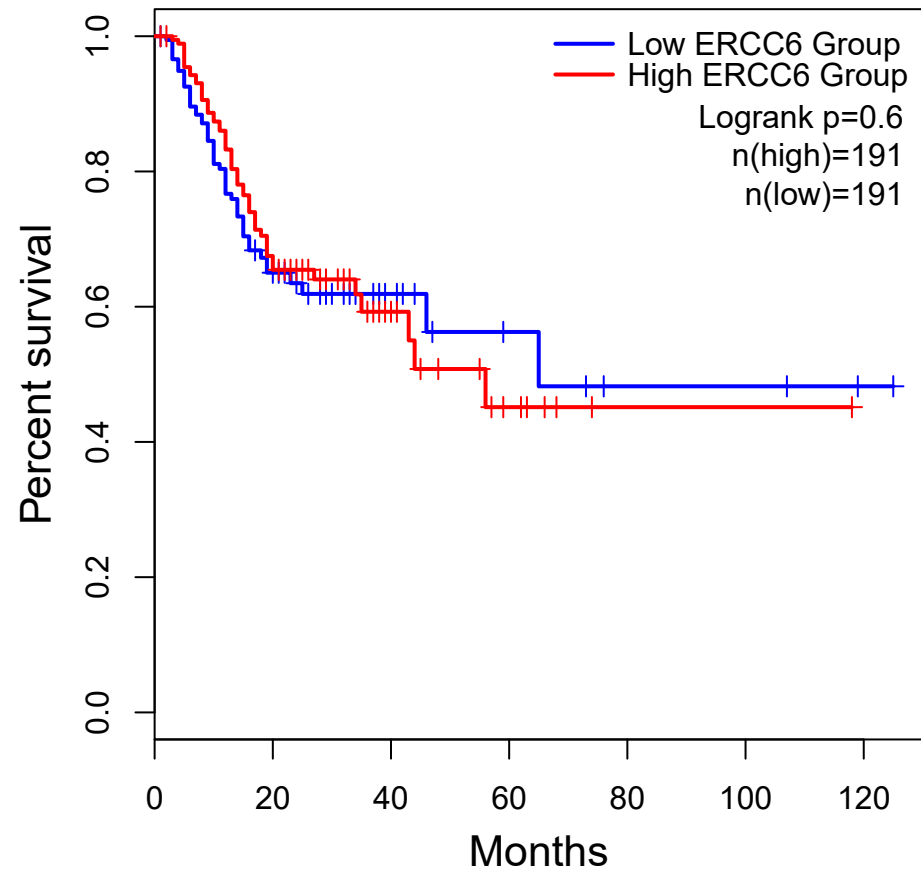

A

# Disease Free Survival

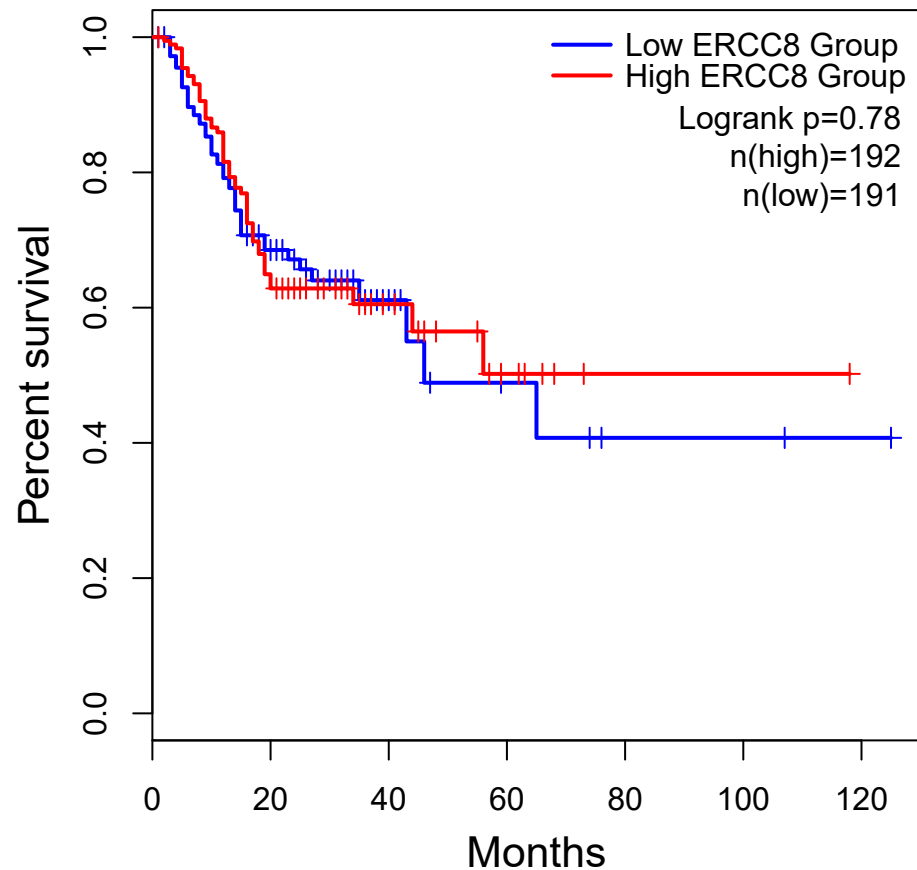

B

Supplement: Supplemental Information 4 — A.ERCC6; B.ERCC8 [file peerj-09-11791-s004.pdf]

# PI3K-AKT SIGNALING PATHWAY

ERCC6/8 related genes

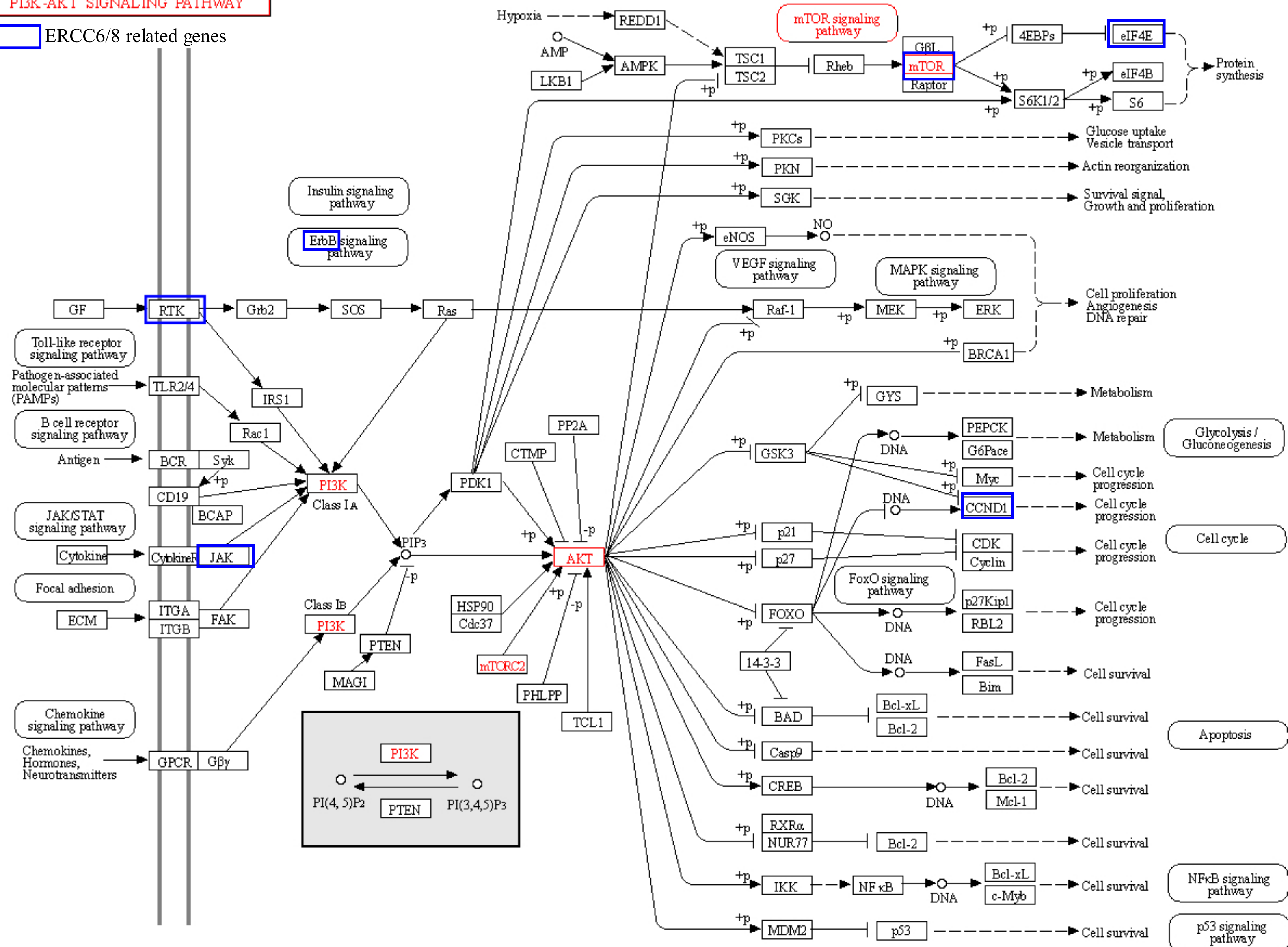

Supplement: Supplemental Information 5 — Source credit: Kanehisa M, Sato Y, Kawashima M, Furumichi M, Tanabe M. KEGG as a reference resource for gene and protein annotation. Nucleic acids research. 2016;44(D1):D457-62. [file peerj-09-11791-s005.pdf]
